# Prediction model for 30-day morbidity after gynecological malignancy surgery

**DOI:** 10.1371/journal.pone.0178610

**Published:** 2017-06-01

**Authors:** Seung-Hyuk Shim, Sun Joo Lee, Meari Dong, Jung Hwa Suh, Seo Yeon Kim, Ji Hye Lee, Soo-Nyung Kim, Soon-Beom Kang, Jayoun Kim

**Affiliations:** 1 Department of Obstetrics and Gynecology, Konkuk University School of Medicine, 120 Neungdong-ro, Gwangjin-gu, Seoul, Korea; 2 Research Coordinating Center, Konkuk University Medical Center, 120 Neungdong-ro, Gwangjin-gu, Seoul, Korea; Indiana University School of Medicine, UNITED STATES

## Abstract

**Objective:**

The potential risk of postoperative morbidity is important for gynecologic cancer patients because it leads to delays in adjunctive therapy and additional costs. We aimed to develop a preoperative nomogram to predict 30-day morbidity after gynecological cancer surgery.

**Methods:**

Between 2005 and 2015, 533 consecutive patients with elective gynecological cancer surgery in our center were reviewed. Of those patients, 373 and 160 patients were assigned to the model development or validation cohort, respectively. To investigate independent predictors of 30-day morbidity, a multivariate Cox regression model with backward stepwise elimination was utilized. A nomogram based on this Cox model was developed and externally validated. Its performance was assessed using the concordance index and a calibration curve.

**Results:**

Ninety-seven (18.2%) patients had at least one postoperative complication within 30 days after surgery. After bootstrap resampling, the final model indicated age, operating time, and serum albumin level as statistically significant predictors of postoperative morbidity. The bootstrap-corrected concordance index of the nomogram incorporating these three predictors was 0.656 (95% CI, 0.608–0.723). In the validation cohort, the nomogram showed fair discrimination [concordance index: 0.674 (95% CI = 0.619–0.732] and good calibration (*P* = 0.614; Hosmer-Lemeshow test).

**Conclusion:**

The 30-day morbidity after gynecologic cancer surgery could be predicted according to age, operation time, and serum albumin level. After further validation using an independent dataset, the constructed nomogram could be valuable for predicting operative risk in individual patients.

## Introduction

In 2014, an estimated 94,990 gynecologic cancer cases, including uterine corpus, ovarian, and cervix cancers, were diagnosed in the USA [1]. In Korea, the incidence of gynecological cancer was over 7,400 cases in 2010 [2]. For the vast majority of gynecological cancer patients, the primary treatment was surgery. Depending on the primary site and the extent of the surgical procedures, several studies have estimated morbidity rates after gynecologic cancer surgeries to range from 13% and 86% [3–5].

Postoperative morbidity is an undesirable but critical issue for both clinicians and gynecologic cancer patients. It may cause delays in subsequent chemotherapy or radiotherapy. Furthermore, it considerably escalates the cost of postoperative management [6]. Thus, it is important to preoperatively identify the subgroup that has a higher risk of morbidity after gynecologic cancer surgery.

Previous studies have identified potential risk factors (age, operation time, anemia, co-morbidities, physical status, surgical site and type of surgical procedure, among other factors) for postoperative morbidity in non-gynecologic surgery [7]. Several prediction models using the identified risk factors have been suggested to forecast the occurrence of postoperative morbidity in general surgery [8–10], cardiac surgery [11], esophageal cancer surgery [12], and hepatocelluar cancer surgery [13]. However, the individualized prediction models for morbidity after gynecologic cancer surgery are relatively rare [14,15].

Due to the lack of an effective tool to estimate individual risk for postoperative morbidity in the field of gynecologic cancer, this study aimed to develop and validate a nomogram that could predict 30-day morbidity after gynecologic cancer surgery.

## Materials and methods

### Patients

This study was approved by the institutional review board of the Center (KUH1040040). The enrolled patients were obtained from a computerized database of gynecologic cancer cases at Konkuk University Hospital between May 2005 and December 2015. Data were accessed anonymously. The inclusion criteria were as follows: pathologically confirmed gynecological (cervix, corpus, ovarian/tubal/peritoneal, vulva, or vaginal) cancer; received elective surgery at our institution; and received postoperative care at our institution. Those referred for a re-staging procedure after an initial surgery at an outside hospital were also included. The patients who received postoperative care at other hospitals and/or had operations for recurrent tumors were excluded.

### Variables

The clinicopathological and follow-up data were retrospectively reviewed from the electronic medical records. Variables for analysis were as follows: age; parity; body mass index (BMI); consumption of alcohol > 2 standard drinks per day [14]; current smoker; physical status according to the American Society of Anesthesiologist (ASA) classification system; comorbidity status; history of chemotherapy or radiotherapy related to the disease prior to surgery; pre-operative lab results, including serum albumin, glutamic oxaloacetic transaminase (SGOT), glutamic pyruvic transaminase (SGPT), hematocrit, platelet count, prothrombin time (PT), and activated partial thromboplastin time (aPTT); primary pathology; International Federation of Gynecology and Obstetrics (FIGO) stage; extent of surgical procedure; type of surgical approach; operation time (initial incision to skin closure); and estimated blood loss (EBL). Comorbidity was expressed as a numerical value using the Charlson comorbidity index (CCI) (Table 1) [16]. Based on the number and complexity of the each surgical procedure, the extent of surgery was categorized into three groups (low, intermediate, high) using a surgical complexity score (SCS) [17] (S1 Table). This parameter was introduced by Aletti et al., and since 2007, it has been rigorously validated and used as a surrogate indicator for surgical complexity [17] [14,15,18]. Therefore, we believe that the effect of heterogeneous surgical procedures is minimized.

**Table 1:** Characteristics of the model development and validation cohorts.

Characteristics		Model development cohort (n = 373)	Validation cohort (n = 160)	*P*
Age, years	Median (range)	49 (14–81)	52 (18–76)	0.057^b^
BMI, kg/m^2^	Median (range)	23.5 (16.5–43.1)	23.8 (14.2–38.1)	0.963^b^
Parity, n	Median (range)	2 (0–7)	2 (0–7)	0.575^b^
Alcohol; >2 standard drinks/day, n (%)		10 (2.7)	8 (5.0)	0.174^c^
Current smoker, n (%)		13 (3.5)	4 (2.5)	0.553^c^
ASA physical status score, n (%)				0.867^c^
1		113 (30.5)	55 (34.4)	
2		222 (60.0)	80 (50.0)	
3		35 (9.5)	25 (15.6)	
Charlson comorbidity index^a^	Median (range)	0 (0–5)	0 (0–5)	0.063^b^
Coronary artery disease 1, n (%)		6 (1.6)	1 (0.6)	
Congestive heart failure 1, n (%)		2 (0.5)	2 (1.3)	
Cerebrovascular disease 1, n (%)		6 (1.6)	4 (2.5)	
Peripheral vascular disease 1, n (%)		2 (0.5)	2 (1.3)	
Hypertension 1, n (%)		77 (20.6)	26 (16.3)	
Dementia 1, n (%)		1 (0.3)	1 (0.6)	
Diabetes (mild or moderate) 1, n (%)		31 (8.3)	10 (6.3)	
Pulmonary disease 1, n (%)		16 (4.3)	14 (8.8)	
Renal disease 1, n (%)		5 (1.3)	5 (3.1)	
Any prior malignant tumor 2, n (%)		20 (5.4)	9 (5.6)	
Hepatic disease 3, n (%)		15 (4.0)	11 (6.9)	
Prior chemotherapy/radiotherapy, n (%)		24 (6.4)	15 (9.4)	0.232^c^
Preoperative systemic infection, n (%)		7 (1.9)	4 (2.5)	0.741ex
Referred for restaging, n (%)		51 (13.7)	23 (14.4)	0.830^c^
Primary pathology, n (%)				0.053^d^
Cervix		130 (34.9)	38 (23.8)	
Corpus		90 (24.1)	52 (32.5)	
Ovary		150 (40.2)	68 (42.6)	
Vulva/Vaginal		3 (0.8)	2 (1.3)	
Albumin, g/dL	Median (range)	4.2 (1.8–5.1)	4.2 (2.3–5.1)	0.771^b^
Hematocrit	Median (range)	37.4 (23–47)	38.1 (29–46)	0.047^b^
Platelet, 10^3^/mm^3^	Median (range)	255 (86–574)	251 (112–496)	0.594^b^
SGOT, U/L	Median (range)	21 (11–80)	22 (13–61)	0.675^b^
SGPT, U/L	Median (range)	17 (4–78)	16 (6–54)	0.404^b^
PT, INR	Median (range)	1.00 (0.85–1.77)	1.01 (0.88–1.19)	0.941^b^
aPTT, sec	Median (range)	35.2 (24.6–61.6)	34.8 (21.3–55.9)	0.035^b^

Abbreviations: BMI, body mass index; ASA, American Society of Anesthesiology; SGOT, serum glutamic oxaloacetic transaminase; SGPT: serum glutamic pyruvic transaminase; PT, prothrombin time; aPTT, activated partial thromboplastin time.

^a ^Charlson comorbidity index was calculated according to the scoring system established by Charlson et al. [16].

^b ^Mann–Whitney *U* test.

^c ^Pearson's chi-squared test.

^d ^Fisher's exact test.

### Statistical analysis

All postoperative adverse events were recorded in accordance with the American College of Surgeons’ National Surgical Quality Improvement Program (NSQIP) [19]. The primary outcome was postoperative morbidity within 30 days after surgery. Having one or more of the following adverse events was defined as major morbidity (Table 2) [3,8,15,19]: pneumonia, wound disruption, deep or organ-space surgical site infection, unplanned intubation, pulmonary embolism, on ventilator > 48 hours, renal failure requiring dialysis, any urinary tract injury (fistula, obstruction or leak), stroke/cerebrovascular accident, coma >24 hours, peripheral nerve injury, cardiac arrest requiring cardiopulmonary resuscitation, myocardial infarction, bleeding requiring>4 U of transfused blood, any bowel injury (leak, fistula), prolonged ileus (>6 days), deep venous thrombosis, sepsis, unplanned readmission, unplanned reoperation, and postoperative death. Superficial surgical site infections or urinary tract infections were not considered major morbidities.

**Table 2:** Surgical outcomes and postoperative morbidity.

Characteristics		Model development cohort (n = 373)	Validation cohort (n = 160)
Operation time, min	Median (range)	213 (35–495)	215 (54–620)
Estimated blood loss, mL	Median (range)	500 (50–8000)	500 (40–7000)
Postoperative morbidity, n (%)		69 (18.5)	28 (17.5)
Bleeding requiring>4 U of transfused blood		25 (6.7)	12 (7.5)
Sepsis		3 (0.8)	2 (1.3)
Pneumonia		11 (2.9)	5 (3.1)
Pulmonary embolus or deep venous thrombosis		5 (1.3)	2 (1.3)
Any type of complication requiring reoperation		7 (1.9)	4 (2.5)
Any bowel injury (leak, fistula, anastomotic leakage)		4 (1.1)	1 (0.6)
Prolonged ileus (>6 days)		15 (4.0)	6 (3.8)
Urinary tract injury (ureteral fistula, obstruction or leak),		9 (2.4)	4 (2.5)
On ventilator >48 h after operation		3 (0.8)	1 (0.6)
Renal failure requiring dialysis		3 (0.8)	1 (0.6)
Myocardial infarction		2 (0.5)	1 (0.6)
Stroke/cerebrovascular accident		1 (0.3)	1 (0.6)
Unplanned intubation		6 (1.6)	2 (1.3)
Wound disruption		29 (7.8)	10 (6.3)
Deep or organ-space surgical site infection		7 (1.9)	2 (1.3)
Coma >24 hours		1 (0.3)	0
Peripheral nerve injury		2 (0.5)	1 (0.6)
Cardiac arrest requiring cardiopulmonary resuscitation		0	0
Unplanned readmission <30days		13 (3.5)	7 (4.4)
Death <30 days		2 (0.5)	0

Grouping of categorical variables was done before analysis. Age, BMI, parity, EBL, serum albumin, GOT, GPT, hematocrit, platelet count, PT, aPTT, and operative time were considered continuous variables. Alcohol consumption, current smoker, ASA physical status score, CCI, history of chemotherapy or radiotherapy before surgery, referral for restaging, SCS, type of surgical approach, and primary pathology were considered categorical variables.

The nomogram was built as previously described [18]. To develop a robust and well-calibrated nomogram to predict postoperative morbidity, a logistic regression model was built using a development cohort of 373 patients and validated with a cohort of 160 patients. First, the bivariate relationship between risk factors and postoperative morbidity was assessed. Next, the predictive values obtained by univariate analyses (*P* < 0.2) were tested by bootstrap resampling in which a logistic regression model with a backward elimination procedure included 1,000 repetitions. The criterion for inclusion of predictors in the final logistic model was a 50% relative frequency of selection by bootstrap resampling. We accounted for missing values by multiple imputation [20]. To assess the model fit, the concordance index was used to measure discrimination by calculating the area under the receiver operating characteristics curve. The Hosmer-Lemeshow test was used to assess calibration. The model was applied to the validation cohort for external validation. Using the same methods, the discrimination and model calibration were tested. All analyses were performed using SPSS (version 19.0; SPSS, Chicago, IL) and R version 3.0.0 (http://cran.r-project.org/mirrors.html). In this study, *P* < 0.05 was to be considered significant.

## Results

### Patient characteristics

During the study period, 739 patients were newly diagnosed with gynecologic cancer and treated at our institution. Of these patients, 558 underwent elective surgery. In total, 533 patients met all of the inclusion criteria. Before analysis, patients were allocated at a 7:3 ratio to either the model development (n = 373; May 2005−June 2013) or validation cohort (n = 160; July 2013– December 2015) (S1 Fig). The characteristics of the model development and validation cohorts are summarized in Table 1. There were no significant differences between the age, BMI, parity, proportion of patients smoking, history of neoadjuvant chemotherapy or radiotherapy, referral for restaging, pre-operative lab results, primary pathology, and CCI in the two cohorts.

### Surgical procedures and outcomes

The most frequent surgical procedures were hysterectomy and/or salpingo-oophorectomy followed by pelvic lymphadenectomy (Table 3). Of the entire cohort, 112 (21%) patients were categorized into the intermediate complex surgery group by SCS and 144 (27%) patients had a laparoscopic procedure.

**Table 3:** Surgical procedures and related factors.

	Model development cohort (n = 373)	Validation cohort (n = 160)	*P*
Surgical procedures; SCS, n (%)			
Hysterectomy-BSO; 1	341 (91.4)	147 (91.9)	
Omentectomy; 1	113 (30.2)	55 (34.4)	
Pelvic lymphadenectomy; 1	263 (70.5)	110 (68.7)	
Para-aortic lymphadenectomy; 1	110 (29.5)	52 (32.5)	
Pelvic peritoneum stripping; 1	16 (4.3)	13 (8.1)	
Abdominal peritoneum stripping; 1	17 (4.6)	6 (3.7)	
Small bowel resection; 1	21 (5.6)	11 (6.9)	
Large bowel resection; 2	22 (5.9)	11 (6.9)	
Liver resection; 2	2 (0.5)	2 (1.3)	
Splenectomy; 2	7 (1.9)	4 (2.5)	
Diaphragm stripping; 2	6 (1.6)	5 (3.1)	
Recto-sigmoidectomy T-T anastomosis; 3	18 (4.8)	11 (6.9)	
Surgical complexity score group, n (%)			0.912^a^
SCS ≤ 3: Low complex surgery.	284 (76.1)	118 (73.7)	
SCS 4–7: Intermediate complex surgery.	77 (20.7)	35 (21.9)	
SCS ≥ 8: High complex surgery.	12 (3.2)	7 (4.4)	
Type of surgical approach, n (%)			0.098^b^
Laparotomy	280 (75.1)	109 (68.1)	
Laparoscopy	93 (24.9)	51 (31.9)	

Abbreviations: BSO, bilateral salpingoophorectomy; SCS, surgical complexity score.

^a ^Fisher's exact test.

^b ^Pearson's chi-squared test.

The 30-day postoperative morbidity incidence rate for both the model development and validation cohorts was 18.2% (69/373 and 28/160, respectively). Further details regarding the overall morbidity are shown in Table 2. The most common morbidity was wound disruption (39/533 = 7.3%), followed by bleeding requiring>4 U of transfused blood (37/533 = 6.9%), prolonged ileus (21/533 = 3.9%) and unplanned readmission (20/533 = 3.8%).

### Model development for the prediction of 30-day postoperative morbidity

**Fig 1:**
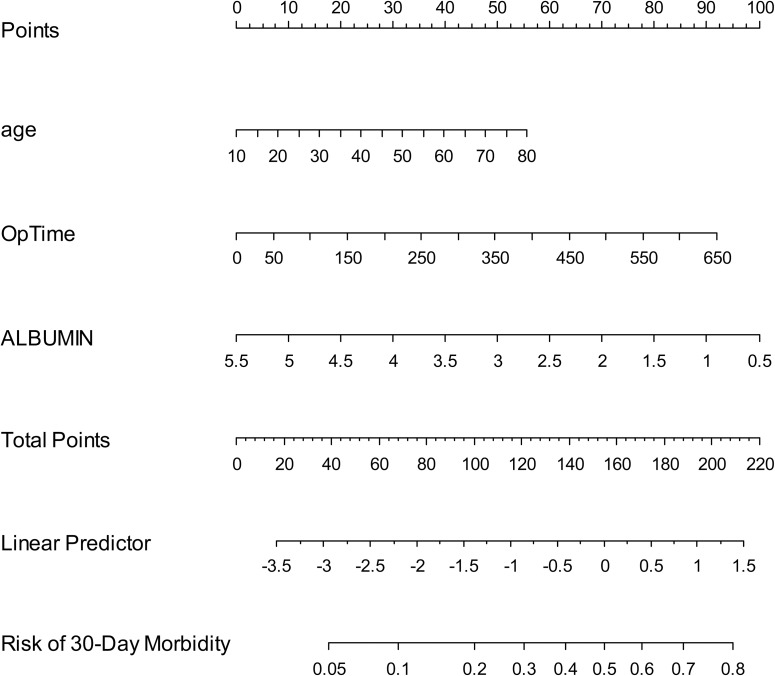
Nomogram that can predict 30-day morbidity after gynecological cancer surgery. This nomogram incorporates three variables. For each level of each prognostic variable, points were allocated according to the scale shown here. The total score was determined by adding individual parameter points and was used to calculate the predicted probability of 30-day morbidity. A total score of 155 was assigned a value of 0.5 and used to define the groups at high-risk of 30-day morbidity after gynecological malignancy surgery.

**Fig 2:**
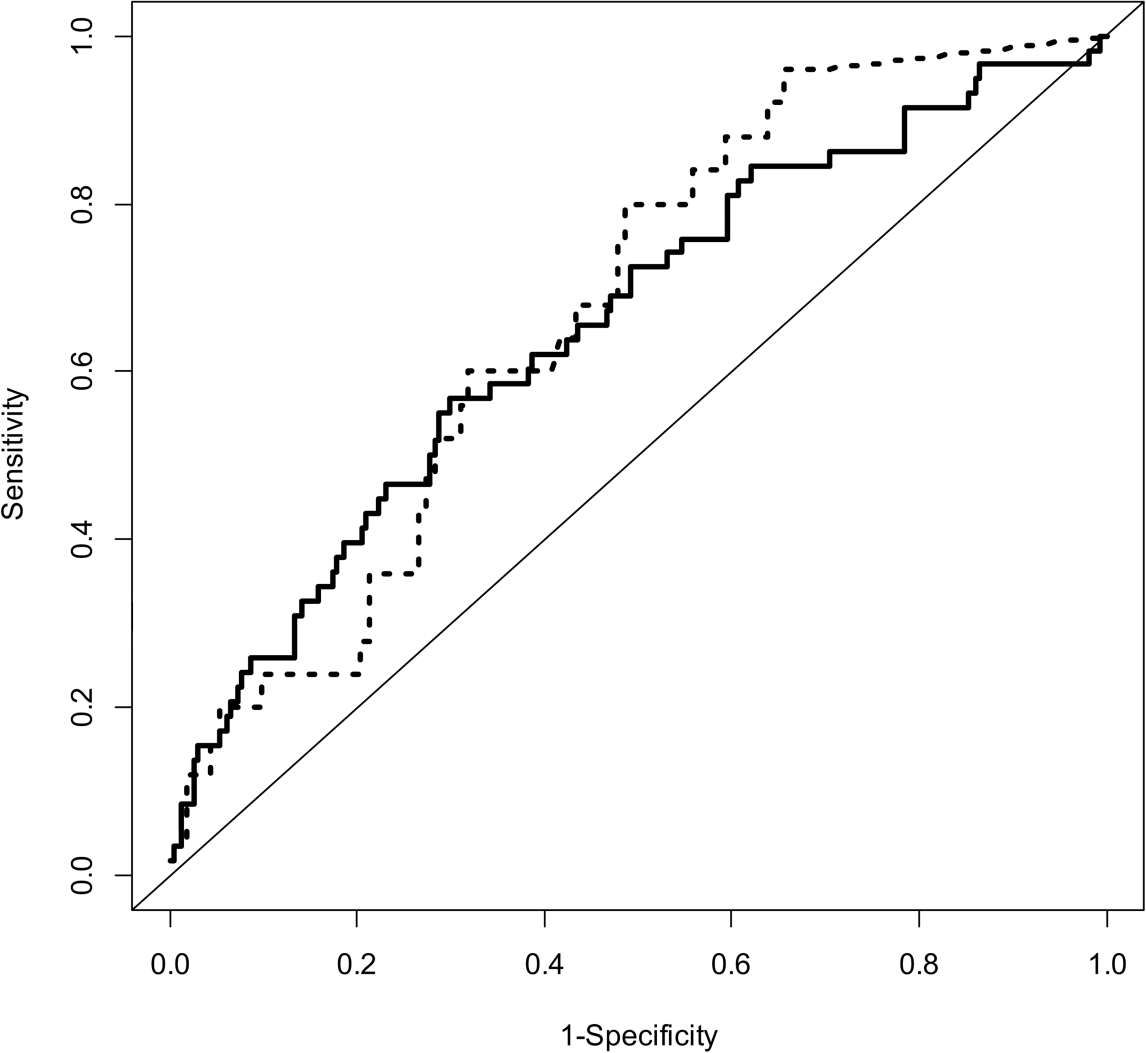
Performance of the nomogram for predicting 30-day morbidity after gynecological cancer surgery. After 1000 repetitions, the bootstrap-corrected concordance index of the model development cohort (solid line) was 0.656 (95% CI = 0.608–0.723). In the validation cohort (dashed line), the bootstrap-corrected concordance index was 0.674 (95% CI = 0.619–0.732).

Table 4 shows the results of the logistic regression analyses used to identify predictors of 30-day postoperative morbidity. After bootstrap resampling, the final model indicated that age, operation time, and serum albumin level were statistically significant predictors. The coefficients of age, operating time and serum albumin level were 0.0202, -0.0036 and -0.5086, respectively. These coefficients led to the following prediction model for postoperative morbidity: logit (morbidity) = [-1.3386 + 0.0202 * (age) + 0.00360 * (operating time) - 0.5086 * (serum albumin level)]. A nomogram was constructed based on this logistic regression model (Fig 1). The point value assigned to each factor was proportional to the odds ratio derived from the beta coefficients for each factor determined by the regression analysis. After 1000 repetitions, the bootstrap-corrected concordance index of the model was 0.656 (95% CI = 0.608–0.723) (Fig 2). In the validation cohort, the discrimination accuracy of the model was 0.674 (95% CI = 0.619–0.732). The Hosmer-Lemeshow test yielded a *P* value of 0.774 for the model development cohort, which indicates that the nomogram had a good fit. For the validation cohort, the nomogram also fit the data (*P* = 0.614; Hosmer-Lemeshow test).

**Table 4:** Univariate and multivariate logistic regression analyses for predicting 30-day morbidity after gynecological cancer surgery.

Variables		Univariate analysis		Multivariate analysis	
		Odds ratio (95% CI)	*P*	Odds ratio (95% CI)	*P*
Age, years ^a^		1.024 (1.002–1.048)	0.0359	1.020 (1.002–1.044)	0.031
BMI, kg/m^2^ ^a^		1.004 (0.946–1.064)	0.907		
Parity, n ^a^		1.047 (0.865–1.267)	0.640		
Alcohol; >2 standard drinks/day	Yes	0.751 (0.165–3.421)	0.711		
Current smoker	Yes	0.316 (0.041–2.439)	0.269		
ASA physical status score	1	1			
	2	1.202 (0.633–2.281)	0.8309		
	3	1.648 (0.639–4.246)	0.3501		
Preoperative systemic infection	Yes	2.319 (0.568–9.467)	0.241		
Charlson comorbidity index	0	1			
	1	1.325 (0.674–2.605)	0.1911		
	> = 2	0.656 (0.261–1.658)	0.2311		
Prior chemotherapy/radiotherapy	Yes	1.566 (0.545–4.496)	0.4045		
Referred for restaging	Yes	1.163 (0.526–2.571)	0.7085		
Primary pathology	Cervix	1			
	Corpus	1.491 (0.681–3.261)	0.6943		
	Ovary	1.714 (0.851–3.454)	0.2447		
Type of surgical approach	Laparotomy	1			
	Laparoscopy	0.289 (0.111–0.753)	0.0111		
SCS	Low	1			
	Intermediate+High	2.157 (1.169–3.979)	0.0139		
Operation time, min ^a^		1.004 (1.001–1.008)	0.0053	1.004 (1.001–1.007)	0.0263
EBL, mL ^a^		1.000 (1.000–1.001)	0.0319		
Albumin, g/dL ^a^		0.503 (0.310–0.818)	0.0056	0.601 (0.369–1.004)	0.0516
Hematocrit a		1.061 (0.982–1.146)	0.1324		
Platelet, 10^3^/mm^3^ ^a^		1.001 (0.996–1.003)	0.8447		
SGOT, U/L ^a^		1.013 (0.984–1.043)	0.3754		
SGPT, U/L ^a^		1.017 (0.992–1.042)	0.1908		
PT, INR ^a^		1.114 (0.071–17.523)	0.939		
aPTT, sec ^a^		1.019 (0.954–1.089)	0.5774		

Abbreviations: BMI, body mass index; ASA, American Society of Anesthesiology; SCS, surgical complexity score; EBL, estimated blood loss; SGOT, serum glutamic oxaloacetic transaminase; SGPT: serum glutamic pyruvic transaminase; PT, prothrombin time; aPTT, activated partial thromboplastin time; CI, confidence interval.

^a^ as continuous variable.

### Laparotomy vs. laparoscopy

As the surgical outcomes for laparoscopy are different from those of laparotomy, a separate analysis was performed for the two subgroups stratified by surgical approaches (laparotomy vs laparoscopy). Of 389 (73.0%) patients who underwent laparotomy, 84 patients (21.6%) had 30-day postoperative morbidity. The results of the univariate and multivariate binary logistic regression for this cohort are summarized in S2 Table. The multivariate analysis revealed that age, operation time, and serum albumin level were statistically significant predictors. This result was similar to the results of the model development cohort. When we tested the constructed model for the cohort who underwent laparotomy, the concordance index of the constructed model was 0.681 (95% CI = 0.616–0.747), and the Hosmer-Lemeshow test yielded a *P* value of 0.653. Of 144 (27.0%) patients who underwent laparoscopy, 13 patients (8.9%) had 30-day postoperative morbidity. After univariate binary logistic regression, operation time was the only significant predictor of 30-day postoperative morbidity in this cohort of patients. Therefore, further multivariate analysis was not performed in this cohort. When we tested the constructed model using the laparoscopy cohort, the concordance index of the model was 0.793 (95% CI = 0.656–0.930), and the Hosmer-Lemeshow test yielded a *P* value of 0.080. Therefore, our constructed prediction model fit well for both the laparoscopy and laparotomy cohorts.

## Discussion

In the present study, a nomogram for predicting 30-day postoperative morbidity after gynecologic cancer surgery was constructed and internally validated. The incorporated factors for the model were age, operating time, and serum albumin level. In terms of model performance, the constructed model showed fair discrimination and good calibration.

The procedures of gynecological cancer surgery are usually complex, and the postoperative morbidity ranges from 18 to 26% [3,14,21]. The 30-day postoperative morbidity in our study was 18%, which is comparable to a previously published incidence. The postoperative morbidity adversely affects gynecologic cancer patients for several reasons, including escalating the costs of health care and increasing stress on a patient and their family [6]. Furthermore, if adjuvant treatment is required, timely installation of chemotherapy or radiotherapy is delayed, which is associated with impaired overall survival [22]. Thus, a preoperative prediction to identify patients at potential risk of postoperative morbidity is critical.

Several prediction models for postoperative morbidity in general surgery, cardiac surgery, and hepatocellular carcinoma populations have been introduced [8,9,13]. However, the aforementioned models cannot reliably be used in gynecological oncology [21,23,24]. Apparently, the risk factors for postoperative morbidity differs based on the surgical sites and the type of procedures [7]. Recently, Clark et al. validated whether the Surgical Apgar Score (SAS) can predict post-operative morbidity in 632 patients undergoing hysterectomies for malignancies. In their study, SAS was not able to predict the postoperative morbidity [21]. Although SAS is a simple surgical outcome score based on easily obtainable intra-operative data (i.e., EBL, mean arterial pressure, and heart rate), many more factors may contribute to an increased risk for morbidity in gynecologic cancer surgery. Likewise, Rivard et al. intended to validate the performance of the NSQIP surgical risk calculator in patients undergoing gynecologic cancer surgery [24]. Using their 1094 patients, the overall performance of the calculator was worse for gynecologic cancer patients than for general surgery patients. Therefore, a tailored prediction model should be developed for this patient population.

Because of one of these efforts, Kondalsamy-Chennakesavan et al. have suggested the risk prediction model for postoperative morbidity using data from 369 patients treated for suspected gynecologic cancer [14]. The concordance index of the model incorporating 4 variables (SCS, ASA score, SGOT and BMI) was 0.7. However, 35% of the enrolled patients were ultimately diagnosed with benign pathology after surgery. Meanwhile, the present study included only pathologically proven gynecological cancer patients.

An interesting feature of our constructed nomogram is the incorporation of the serum albumin level. In the previously suggested models for gynecologic cancer, the serum albumin level had not been analyzed as a potential predictor for postoperative morbidity [14,15]. Serum albumin is universally accepted to be a better index for protein-energy malnutrition than anthropomorphic markers [25]. Preoperative hypoalbuminemia (defined as albumin level <3.5 g/dl) is associated with compromised immunity and impaired tissue healing, which affects surgical site infections and anastomotic leakage as well as remote infections, such as pneumonia [26]. Aletti et al. suggested that serum albumin is an independent predictor of 30-day morbidity in the analysis of 564 patients with advanced stage ovarian cancer [17]. Recently, Uppal et al. analyzed 2110 patients undergoing gynecologic cancer surgery from the NSQIP dataset and found that the group with preoperative hypoalbuminemia was two times as likely to develop at least one postoperative morbidity [3]. Accordingly, nutritional support [27,28] or alternative treatment strategies that postpone a potentially complex surgery should be considered for hypoalbuminemic patients. Protein-energy malnutrition is frequent due to tumor induced-hypercatabolism and feeding difficulty, especially in ovarian cancer with extensive tumor burden. In this case, primary debulking surgery may cause postoperative morbidities and subsequently delay adjuvant chemotherapy, which affects the overall survival negatively [22]. This high-risk population may benefit from neoadjuvant chemotherapy strategy [29,30].

In the present study, ASA scores and elevated SGOT (≥35 U/L), which have been reported to be predictors of postoperative morbidity in other studies [14,15], did not show a significant prognostic value. Substantial debates exist whether the ASA score alone can be a risk-predictor for postoperative morbidity because of its poor interrater consistency and imprecision [31]. While elevated SGOT is an independent predictor of mortality in general and vascular surgery [32], the role of elevated SGOT in gynecological cancer is unclear [33].

Our model was designed as a user-friendly nomogram that allows for a simple graphical quantification of postoperative morbidity probability. It would enhance the prediction of surgical outcome in the clinical practice and provide objective parameters to select specific populations who might benefit from alternative treatment approaches. Moreover, it can also be used as an audit tool for the quality of surgical care to compare the expected rate of postoperative morbidity with the actual rate [34]. In addition, to develop a reliable model, all clinical predictors should be tested for inclusion. The number of enrolled patients in the current study was sufficiently large to test candidate predictors.

We did not develop the model after the stratification of surgical approaches (laparotomy vs laparoscopy); rather, we analyzed them en toto for the following reasons. First, the number of postoperative morbidity cases in the laparoscopy cohort was not sufficient (n = 13) to develop a reliable prediction model only for this cohort. Second, after multivariate analysis, the surgical approach did not remain as an independent predictor, which may have resulted from the low number of events and cases in the laparoscopy cohort. In this case, incorporating the surgical approach into the model does not improve the model performance [3,14,33,35,36]. Laparoscopy has lower rates of 30-day postoperative morbidity; therefore, to observe a difference in the morbidity rate would require a higher number of patients in each group [3]. Although the results of the separate analyses were favorable for testing whether the constructed model could be applied in the laparotomy or laparoscopy cohorts, the applicability of the model for the patients undergoing laparoscopy might be limited using our database.

There are some limitations to the present study. First, underestimation of the morbidity incidence rate may arise because of the retrospective study design. However, all the included patients received postoperative care in our institution, and the data were extracted from full electronic medical records using a standardized format that was installed and audited since 2005 in our institution. This point may minimize the ascertainment bias. Second, although we performed rigorous internal validation, the nomogram still requires external validation by other institutions to gain general applicability. A nomogram for routine practical use should be developed in a general population rather than in a selection of patients treated in specialized hospitals. The specific characteristics of the population (median BMI of 24, more ovarian than endometrial pathology, low ASA score, low SCS, and three-quarters of cases performed via laparotomy) would likely render the nomogram minimally valuable elsewhere. Third, factors concerning the surgeon, such as skill and experience, were not analyzed in the present model. Although postoperative outcomes vary widely across surgeons and hospitals, these factors are difficult to quantify [37]. Thus, if these factors are incorporated into the model, it could show better performance. Fourth, someone may argue that our model may be skewed for only low-risk surgery group because the number of patients with the intermediate- and the high-risk surgery group is low. However, the proportion of the intermediate- and the high-risk surgery group is 21% and 3.6%, respectively, which is comparable to a previously published data. In the study by Gerestein et al. the proportion of the intermediate- and the high-risk surgery group is 10.9% and 0.7%, respectively [14,15]. Finally, the concordance index is less than 0.7, indicating the weakness of the performance of our model. However, in the field of gynecologic malignancy surgery, the published prediction systems for morbidity tend to have concordance indexes <0.7; 0.61 in the study by Erekson [38], 0.65 in the study by Kondalsamy-Chennakesavan [33], and 0.68 in the study by Gerestein [15]. Therefore, the concordance index of our model is comparable and acceptable. In addition, our study is meaningful because it is the first study to target the Asian population, there can be differences in outcomes between races [15].

## Conclusions

We developed and internally validated a nomogram for predicting the individualized risk of 30-day morbidity after gynecological cancer surgery. This nomogram would be much better served by embracing minimally invasive approaches to surgery. Further tests using a prospective multicenter study with a high number of minimally invasive surgeries are required.

## Supporting information

S1 Table Surgical complexity scoring system based upon complexity and number of surgical procedures performed. (DOCX)

S2 Table Univariate and multivariate logistic regression analyses for predicting 30-day morbidity after gynecological cancer surgery: Laparotomy cases. (DOC)

S1 Fig Flow chart illustrating patient inclusion. (TIFF)

S1 File All relevant data anonym. (SAV)
