# Supplementary material for: Prediction model for 30-day morbidity after gynecological malignancy surgery
Source: PLoS One. 2017 Jun 1;12(6):e0178610. doi: 10.1371/journal.pone.0178610 (PMC5453555; doi:10.1371/journal.pone.0178610)
Supplement: S1 Table — (DOCX) [file pone.0178610.s001.docx]

**S1 Table.** Surgical complexity scoring system based upon complexity and number of surgical procedures performed

| *Procedure* | *Points* |
| --- | --- |
| Hystrectomy-bilateralsalpingoophrectomy | 1 |
| Omentectomy | 1 |
| Pelvic lymphadenectomy | 1 |
| Para-aortic lymphadenectomy | 1 |
| Pelvic peritoneum stripping | 1 |
| Abdominal peritoneum stripping | 1 |
| Recto-sigmoidectomy _ T–T anastomosis | 3 |
| Large bowel resection | 2 |
| Diaphragm stripping/resection | 2 |
| Splenectomy | 2 |
| Liver resection/s | 2 |
| Small bowel resection/s | 1 |
| *Complexity score groups* |  |
| 1 (low) | ≤ 3 |
| 2 (intermediate) | 4–7 |
| 3 (high) | ≥ 8 |
