# Supplementary material for: Prediction model for 30-day morbidity after gynecological malignancy surgery
Source: PLoS One. 2017 Jun 1;12(6):e0178610. doi: 10.1371/journal.pone.0178610 (PMC5453555; doi:10.1371/journal.pone.0178610)
Supplement: S2 Table — (DOC) [file pone.0178610.s002.doc]

**S2 Table.** Univariate and multivariate logistic regression analyses for predicting 30-day morbidity after gynecological cancer surgery: laparotomy cases.

| Variables |  | Univariate analysis | | Multivariate analysis | |
| --- | --- | --- | --- | --- | --- |
|  |  | Odds ratio (95% CI) | *P* | Odds ratio (95% CI) | *P* |
| Age, years ^a^ |  | 1.020 (1.001–1.040) | 0.040 | 1.023 (1.003–1.044) | 0.027 |
| BMI, kg/m^2^ ^a^ |  | 1.019 (0.959–1.082) | 0.548 |  |  |
| Parity, n ^a^ |  | 1.014 (0.828–1.242) | 0.892 |  |  |
| Alcohol; >2 standard drinks/day | Yes | 0.490 (0.110–2.187) | 0.350 |  |  |
| Current smoker | Yes | 0.281 (0.036–2.182) | 0.225 |  |  |
| ASA physical status score | 1 | 1 |  |  |  |
|  | 2 | 0.805 (0.466–1.391) | 0.437 |  |  |
|  | 3 | 1.600 (0.780–3.282) | 0.200 |  |  |
| Preoperative systemic infection | Yes | 1.637 (0.414–6.467) | 0.482 |  |  |
| Charlson comorbidity index | 0 | 1 |  |  |  |
|  | 1 | 1.075 (0.601–1.923) | 0.808 |  |  |
|  | >=2 | 0.671 (0.330–1.365) | 0.271 |  |  |
| Prior chemotherapy/radiotherapy | Yes | 0.969 (0.429–2.193) | 0.941 |  |  |
| Referred for restaging | Yes | 1.041 (0.546–1.986) | 0.903 |  |  |
| Primary pathology | Cervix | 1 |  |  |  |
|  | Corpus | 1.382 (0.684–2.792) | 0.368 |  |  |
|  | Ovary | 1.778 (0.994–3.180) | 0.052 |  |  |
| SCS | Low | 1 |  |  |  |
|  | Intermediate+High | 4.670 (2.827–7.715) | 0.041 |  |  |
| Operation time, min ^a^ |  | 1.004 (1.001–1.006) | 0.017 | 1.003 (1.001–1.006) | 0.036 |
| EBL, mL ^a^ |  | 1.000 (1.000–1.001) | 0.019 |  |  |
| Albumin, g/dL ^a^ |  | 0.599 (0.394–0.910) | 0.016 | 0.649 (0.420–1.003) | 0.052 |
| Hematocrit a |  | 1.028 (0.966–1.094) | 0.383 |  |  |
| Platelet, 10^3^/mm^3^ ^a^ |  | 1.001 (0.997–1.003) | 0.937 |  |  |
| SGOT, U/L ^a^ |  | 1.012 (0.987–1.037) | 0.349 |  |  |
| SGPT, U/L ^a^ |  | 1.018 (0.997–1.040) | 0.092 |  |  |
| PT, INR ^a^ |  | 5.546 (0.273–112.858) | 0.265 |  |  |
| aPTT, sec ^a^ |  | 1.012 (0.957–1.071) | 0.665 |  |  |

Abbreviations: BMI, body mass index; ASA, American Society of Anesthesiology; SCS, surgical complexity score; EBL, estimated blood loss; SGOT, serum glutamic oxaloacetic transaminase; SGPT: serum glutamic pyruvic transaminase; PT, prothrombin time; aPTT, activated partial thromboplastin time; CI, confidence interval.

^a^ as continuous variable.
