# Supplementary figures and images for: Prediction model for 30-day morbidity after gynecological malignancy surgery
Source: PLoS One. 2017 Jun 1;12(6):e0178610. doi: 10.1371/journal.pone.0178610 (PMC5453555; doi:10.1371/journal.pone.0178610)

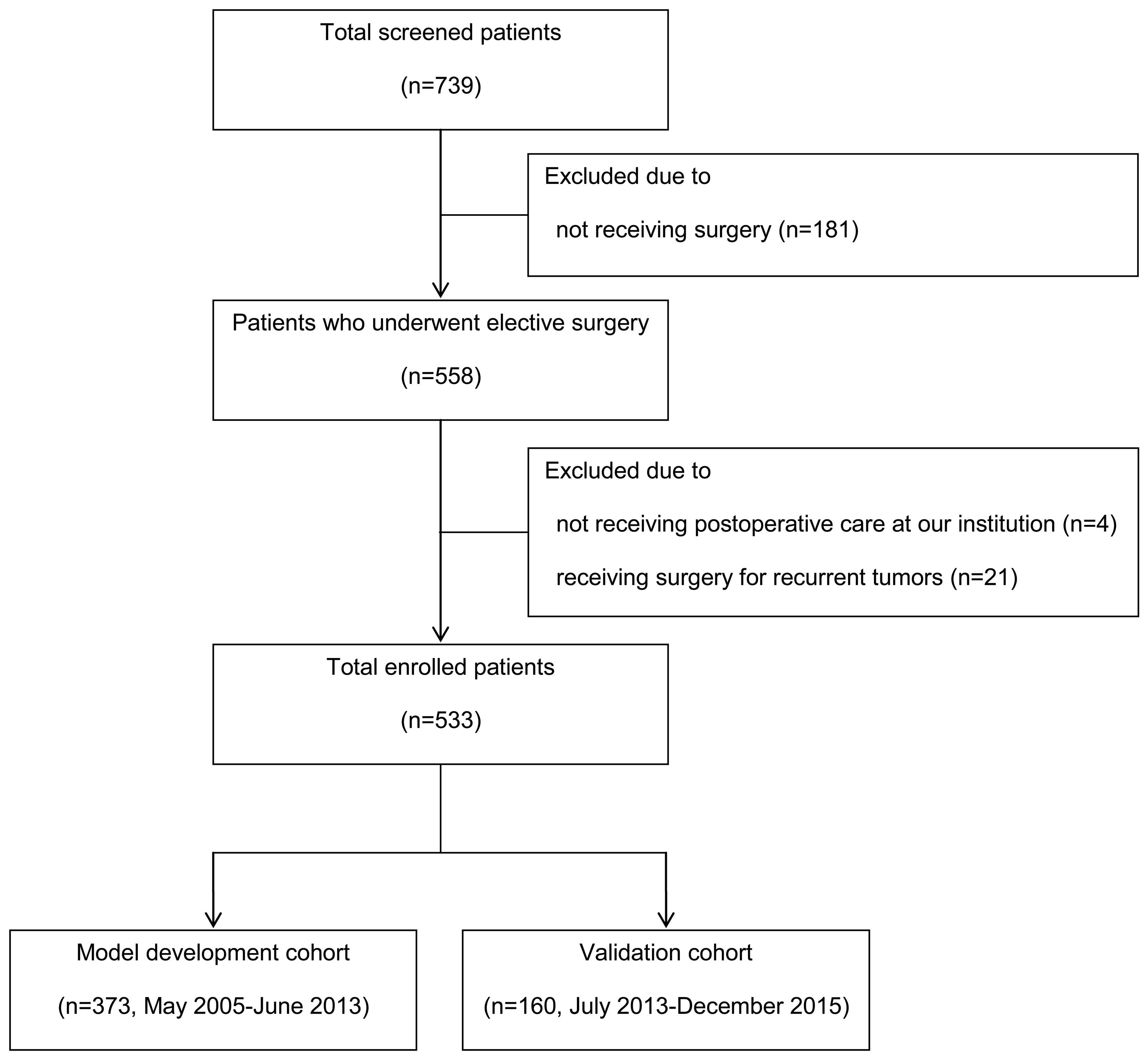

Supplement: S1 Fig — (TIFF) [file pone.0178610.s003.tiff]
